# Emerging infectious disease surveillance using a hierarchical diagnosis model and the Knox algorithm

**DOI:** 10.1038/s41598-023-47010-1

**Published:** 2023-11-13

**Authors:** Mengying Wang, Bingqing Yang, Yunpeng Liu, Yingyun Yang, Hong Ji, Cheng Yang

**Affiliations:** 1 State Key Laboratory of Media Convergence and Communication, Communication University of China, No. 1, Dingfuzhuang East Street, Chaoyang District, Beijing, China; 2 Information Management and Big Data Center, Peking University Third Hospital, No. 49, Huayuan North Road, Beijing, China; 3 Goodwill Hessian Health Technology Co. Ltd, Beijing, China

**Keywords:** Data mining, Software

## Abstract

Emerging infectious diseases are a critical public health challenge in the twenty-first century. The recent proliferation of such diseases has raised major social and economic concerns. Therefore, early detection of emerging infectious diseases is essential. Subjects from five medical institutions in Beijing, China, which met the spatial-specific requirements, were analyzed. A quality control process was used to select 37,422 medical records of infectious diseases and 56,133 cases of non-infectious diseases. An emerging infectious disease detection model (EIDDM), a two-layer model that divides the problem into two sub-problems, i.e., whether a case is an infectious disease, and if so, whether it is a known infectious disease, was proposed. The first layer model adopts the binary classification model TextCNN-Attention. The second layer is a multi-classification model of LightGBM based on the one-vs-rest strategy. Based on the experimental results, a threshold of 0.5 is selected. The model results were compared with those of other models such as XGBoost and Random Forest using the following evaluation indicators: accuracy, sensitivity, specificity, positive predictive value, and negative predictive value. The prediction performance of the first-layer TextCNN is better than that of other comparison models. Its average specificity for non-infectious diseases is 97.57%, with an average negative predictive value of 82.63%, indicating a low risk of misdiagnosing non-infectious diseases as infectious (i.e., a low false positive rate). Its average positive predictive value for eight selected infectious diseases is 95.07%, demonstrating the model's ability to avoid misdiagnoses. The overall average accuracy of the model is 86.11%. The average prediction accuracy of the second-layer LightGBM model for emerging infectious diseases reaches 90.44%. Furthermore, the response time of a single online reasoning using the LightGBM model is approximately 27 ms, which makes it suitable for analyzing clinical records in real time. Using the Knox method, we found that all the infectious diseases were within 2000 m in our case, and a clustering feature of spatiotemporal interactions (*P* < 0.05) was observed as well. Performance testing and model comparison results indicated that the EIDDM is fast and accurate and can be used to monitor the onset/outbreak of emerging infectious diseases in real-world hospitals.

## Introduction

Emerging infectious diseases are defined as “new, emerging, or drug-resistant infectious diseases, the occurrence of which in the population has increased in the past 20 years or for which there are indications that their incidence may increase in the future”^1^. Emerging infectious diseases mainly include new diseases, existing diseases emerging in a new area or population, reintroduced old diseases, previously clinically mild diseases increasing in severity, and previously preventable or treatable diseases becoming uncontrolled or treatment-resistant^2^. Emerging pathogens include bacteria, viruses, parasites, chlamydia, rickettsia, spirochetes, and mycoplasma, among which viruses cause the largest number of emerging infectious disease cases. Emerging infectious diseases, which can cause serious regional or international public health concerns, are one of the most critical public health challenges faced by humankind in the twenty-first century. In the past 30 years, at least 40 emerging infectious diseases have been detected worldwide^1^, and their number is rapidly increasing, posing a considerable threat to human life and health. For example, SARS raged in 2002–2004, the Ebola virus broke out in Africa in 2013–2016, Zika virus cases were detected in 2016, yellow fever was detected in 2016–2018, and so on. Emerging infectious diseases possess the inherent infectivity and prevalence of infectious diseases, they have complex origins, and tracing the sources of these diseases is arduous. Furthermore, they tend to be widespread and difficult to control, making them uniquely uncertain and unpredictable. Owing to these characteristics, these diseases cause extensive harm, as there are no existing guidelines regarding their prevention and control. For example, the coronavirus disease (COVID-19) that occurred in 2019 transmitted rapidly and spread widely, turning into a pandemic, thereby damaging societal and economic stability worldwide^3^. Accordingly, monitoring and early warning of emerging infectious diseases are critical steps towards preventing their spread. Forecasting of emerging infectious diseases and generating an early warning system to avoid their outbreaks are major challenges.

At present, most emerging infectious diseases are discovered because of abnormalities noticed by clinicians. After observation, laboratory biological testing, clinical treatment, and so on, certain disease symptoms are identified and categorized as symptoms of emerging infectious diseases. For example, COVID-19 was discovered when clinicians, who diagnosed the symptoms, reported that many patients were employees of the Huanan Seafood Wholesale Market, and bioinformatics testing showed that the pathogen was a new type of coronavirus^4^. Thus, clinical symptom monitoring and access to the complete medical information of a patient can aid in identifying emerging infectious diseases. Medical institutions are the first line of defense for diagnosing and treating such diseases. Unlike public health monitoring to identify known infectious diseases, timely monitoring of abnormal symptoms and phenomena by medical institutions during infectious disease outbreaks is more beneficial for emerging infectious disease management^5^. Continuous improvements in hospital medical information systems over the years have resulted in the accumulation of a large amount of medical data, including health data, patient information, medical records, inspection records, imaging records, and cost information. In a medical institution with an average daily outpatient volume of 15,000, the medical data volume increases by 50 Gb daily^6^. In general hospitals in China, daily outpatient visits generally exceed 10,000. Therefore, in addition to seeing patients, doctors in medical institutions are expected to identify clues of emerging infectious diseases from many complex and related medical records, and this task is extremely difficult. Furthermore, clinicians need to follow a specific set of rules and regulations while identifying infectious disease risks, performing active identification and legal reporting, and it is objectively difficult for clinicians to take the initiative to provide early warning in the absence of relevant background information. To address these challenges, a big-data-based effective prediction model that can extract features from a large volume of data, perform data mining, and conduct simultaneous time and space monitoring is urgently required.

In general, medical institutions predict the prevalence and outbreak of infectious diseases. Unlike traditional statistical methods, machine learning and deep learning are data driven. To date, several studies on disease diagnosis, hospitalization, prediction of treatment duration, etc. have been conducted using health data obtained from medical institutions^7,8^. These reported studies have revealed several significant theoretical and practical results, which have propelled further research in this field. In recent years, early warning generation and prediction of the onset of infectious diseases have come under the spotlight because of the advances in big data and machine learning; for instance, Lee et al*.*, Feng and Jin, and Wang et al*.* ^9–11^ predicted the onset trend of known infectious diseases. Based on the details, it can be concluded that medical institutions acquire health data during the early stages of the disease as well as are the first points-of-contact for patients infected with various known/unknown infectious diseases. In addition, they have the basic conditions for analyzing the outbreak of emerging infectious diseases. Thus, a combination of machine learning and deep learning will facilitate medical institutions in early detection of the emerging variants of infectious diseases, thereby enabling an effective and timely containment to prevent their outbreak. Thus, this study was designed to construct an emerging infectious disease identification framework based on the real and complete medical records of hospitals, with hierarchical diagnosis model (EIDDM) and the Knox method for spatial cluster as the cores, using machine learning. Considering the accuracy and computational efficiency of data collection in actual medical institutions, a hierarchical diagnosis model, namely the emerging infectious disease detection model (EIDDM), which combines the TextCNN-Attention and LightGBM algorithms, was developed. This proposed framework is suitable for spatial–temporal monitoring of infectious diseases in medical institutions and is anticipated to mitigate the existing key issues related to emerging infectious disease management. The primary objectives and contributions of the present study are as follows:

Analysis of the current research methods on the identification of emerging infectious diseases, and summary of the currently known key issues, including the reported data of symptom events, inadequate reporting of active behavior, and disconnection between non-clinical data (such as online search data) and real medical cases.

Based on the analysis of datasets acquired from multiple medical institutions, structured and unstructured data are applied to various models for separate processing, and a special processing is carried out for different types of features to utilize all the features completely. These processes provide separate datasets for training as well as real-world application of the developed model.

The proposed EIDDM considers both model recognition and online reasoning efficiency. The average emerging-infectious-disease prediction accuracies of the first- and second layer models were 90.47% and 86.93%, respectively. The prediction time of the online reasoning for a single medical record is only 27 ms, which is remarkably less than that required for one-hot encoding of a single medical record in previous studies (68 ms) and is more suitable for real-time scenarios in real clinical medical institutions.

Cluster analysis was performed using the Knox method, which does not require population migration and total population data. A cluster analysis is an effective supplement to EIDDM, which is a hierarchical diagnosis model, to determine the existence of a spatial cluster after discovering the emerging infectious disease cases. This step reduces the misjudgment rate of individual case samples when the model is applied to the data obtained from real medical institutions.

The rest of the paper is organized as follows: Section "Related work" presents and describes the previous studies reported in this field. Section "Materials and methods" discusses the data processing and modeling approaches employed in this study. Section "Results" elucidates the experimental results and compares them with other state-of-the-art research strengths and limitations. Section "Discussions" discusses the rationale for using the one-vs-rest (OvR) strategy to identify emerging infectious diseases. Section "Conclusion" highlights the major conclusions drawn from the findings of this study.

## Related work

Currently, monitoring of emerging infectious diseases is one of the key tasks of public health emergency detection and is accomplished mainly through the collection, analysis, identification, and intervention of the occurrence, spread, and source of infectious diseases in the population. According to Christaki et al. ^12^, infectious disease monitoring is divided into event-based surveillance, web-based real-time surveillance, social media monitoring, and new technologies in pathogen discovery. Event-based surveillance is primarily organized by health authorities. In China, infectious diseases are divided into categories A, B, and C, and doctors in medical institutions are required to report through the national infectious disease reporting system within a limited time ^13^. This system enables medical institutions to act as monitoring sentinels to report and review patients with infectious or suspected infectious diseases. The system also aids health departments and disease control systems in rapidly analyzing and judging any epidemic situation. The observations made in the initial stage of the novel coronavirus epidemic revealed that the system is only limited to daily monitoring and reporting of known infectious diseases. These attributes cannot meet the requirements of information acquisition, early warning, and disposal of emerging infectious diseases ^14^. Furthermore, the elements involved in the surveillance of emerging infectious diseases are diverse and complex, and the fixed indicator combination of traditional disease surveillance is not necessarily applicable to these emerging infectious diseases. Previously, some researchers ^15,16^ conducted surveillance of influenza, influenza-like illnesses, and severe respiratory illnesses based on the symptom surveillance system, which caused a large analysis bias due to the inevitable gap between the symptoms and the actual diagnosis. However, only considering symptoms without incorporating the time and space information cannot satisfy the prediction accuracy of regional infectious disease incidences.

In online-data-based surveillance, the data are chiefly obtained from non-medical institutions in the form of online search engine data, news data, or social data to monitor diseases with potential epidemic risks as well as infectious diseases with seasonal activities. Ref. ^17,18^ report the use of results obtained from web search engines as the data sources for infectious disease symptom monitoring. In these two reported studies, correlation analysis was conducted between the frequency of the related search terms and the actual number of people with symptoms to track the spread of regional infectious diseases. According to Ref. ^19^, Google's prediction of flu trends can be 1 to 2 weeks ahead of that of the Center for Disease Control and Prevention. Juhyeon et al. ^20^ used Medisys to collect internet articles related to infectious diseases to predict the outbreak of infectious diseases using a support vector machine-based model. However, because search engines and news methods require many search queries, in terms of disease coverage, they only support conventional symptoms that the public can describe, such as fever, vomiting, diarrhea, etc., and cannot monitor abnormalities in inspections. In addition, there is a disconnect between the online data and real medical records of medical institutions, and thus, the online method cannot be truly applied in the monitoring scenarios of medical institutions. From the perspective of spatiotemporal monitoring of infectious diseases, online data monitoring facilitates a general large-scale prediction across the country, which cannot be analyzed from the perspective of a spatial cluster. However, a spatial cluster can be located by analyzing the home and work addresses provided in medical records.

With the development of machine learning and neural networks, several methods for early warning and prediction of infectious diseases have been reported to date. Wilkinson et al. ^21^ adopted a statistical process control method, because the data should be independent, and the known parameters should follow a normal distribution. However, the actual transmission mechanism of infectious diseases cannot be completely independent; a certain correlation between the cases is expected. Nevertheless, the machine-learning-based prediction methods exhibit some shortcomings. For instance, although a decision tree can be easily interpreted, a single tree is more sensitive to noise data and has a poor generalization ability. However, an integrated model based on the decision tree can overcome the shortcomings of a single tree. Further, the Bayesian method has a simple logic, is easy to implement, and performs well when the features in the correlation are relatively small. However, the algorithm involves independent assumptions of the feature conditions, and performs poorly when there are many features, and the correlation between the features is large^22^.

Presently, only a few studies on early warning of emerging infectious diseases are available. Li et al. ^23^ extracted the features from historical medical records of various known diseases and constructed a disease probability map. When the probability of a new patient, becoming infected by each known disease type, is less than the threshold set by each known disease, the case of emerging disease types is evident. Although this method considers different diseases independently, it ignores the complexity of the diseases in real medical scenarios. Currently, thousands of common diseases are known, and the relationships among them are complex. Thus, even if the training covers various known diseases, the complex relationship among them has not yet been effectively accounted for by the existing machine-learning-based prediction models. In our previous study ^24^, we used 20,620 real infectious disease datapoints obtained from a large hospital from 2012 to 2022, including outpatient and inpatient sample data, to construct a multi-infectious disease diagnosis model (MIDDM) and obtained 740,000-dimensional feature data, and then performed model training after a sparse data densification processing. In addition, a residual network and an attention mechanism were introduced into the MIDDM to improve the model performance. However, due to the large feature dimension of this method (obtained after the one-hot encoding), the dense expression vector needs to be calculated first through the dense network and finally judged by the classification model. Thus, numerous model parameters are involved in this complicated process. Further, the prediction time has a remarkable impact. The prediction time for a single medical record reaches 68 ms, and the average response time is higher than 400 ms when the number of concurrencies is 100.

Therefore, to realize an effective early warning strategy for emerging infectious diseases via data monitoring, it is necessary to develop an automated monitoring approach that overcomes the issues related to manual monitoring, which involves event and statistical analyses and solely relies on manual reporting of symptoms. In this study, we used a hierarchical model to assess the probability of a single sample being an emerging infectious disease case, and combined the proposed model with the Knox method to analyze clusters. The method considers the case attributes as well as rapidly determines the probability of occurrence of infectious diseases. Further, to achieve data source tracking with high fidelity, it is necessary to utilize the complete existing data of hospitals in combination with real cases for monitoring. In this study, all the medical records obtained from multiple medical institutions were used, and the data time information was retained through time sequencing. To solve the problem of low performance caused by dimension explosion after one-hot coding in the early stage, we adopted the word vector model, word2vec, for data pretraining. Combined with the LightGBM model, word2vec can directly read the word vector in the subsequent model training without the need to calculate the dense vector expression of the current input to improve the prediction efficiency.

## Materials and methods

**Figure 1:**
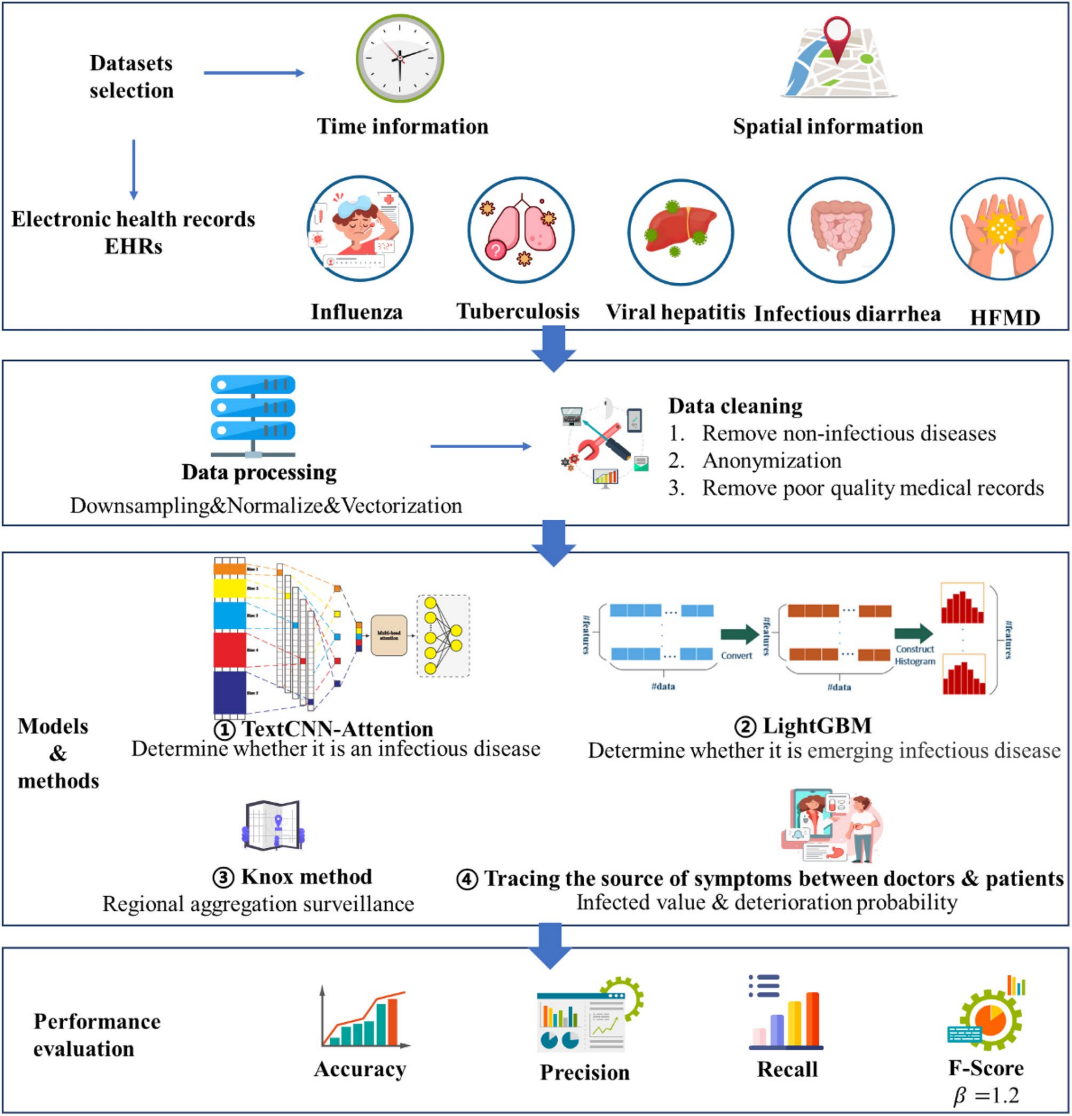
Proposed approach.

This section describes the selection of datasets, data preprocessing, model architecture, classification methods, performance evaluation methods, experimental tools and setup, and validation methods. Figure 1 depicts the artificial intelligence process proposed here for the surveillance of emerging infectious diseases.

### Dataset selection

The data were obtained from five medical institutions in Beijing, China: three in the Haidian District (Headquarters hospital, North hospital, and Party School hospital), one in the Shunyi District, and one in the Daxing District. The location distribution meets the spatial heterogeneityspatial heterogeneity requirement ^25^. ‘Spatial-specific’ refers to characteristics that distinguish things or phenomena in each spatial location from those in other locations. The medical data of five institutions, including all outpatient, emergency, and inpatient data obtained from January 1, 2012 to December 31, 2021 related to the 59 infectious diseases ^26^ highlighted by the China Center for Disease Control and Prevention, are considered. By matching the diagnosis name with the ICD-10 code, all the diseases and sub-diseases belonging to the infectious disease category were included in the dataset, and senior medical experts removed some non-infectious sub-diseases such as thyroid tuberculosis and renal tuberculosis. In order to obtain high-quality training data, names such as diseases and symptoms were standardized and the associated data were verified and subjected to an integrity check. Finally, 37,422 and 9,325,680 cases of infectious and non-infectious diseases, respectively, were identified as listed in Table 1. Some infectious diseases, such as pestis, did not occur, and hence, they are not listed in this table.

**Table 1:** Number of disease categories.

Category of infectious diseases	Number of medical records	Category of infectious diseases	Number of medical records
Non-infectious diseases	56,133	Epidemic hemorrhagic fever	15
Viral hepatitis	23,929	Leprosy	15
Tuberculosis	5313	Melioidosis	12
Influenza	3890	Leptospirosis	12
Hand-foot-and-mouth disease	1573	Anthrax	11
Syphilis	916	Malaria	11
Infectious diarrhea	508	Rift Valley Fever	8
Scarlatina fever	409	Intestinal amoebiasis	20
Schistosomiasis	115	Severe Acute Respiratory Syndrome	7
Measles	97	Melioidosis	7
Typhoid fever	92	Dengue fever	5
COVID-19	87	Filariasis	4
Rubella	81	Epidemic encephalitis B	3
Brucellosis	77	Epidemic cerebrospinal meningitis	3
Epidemic parotitis	66	Visceral leishmaniasis	2
AIDS	47	Epidemic typhus	1
Gonorrhea	40	American trypanosomiasis	1
Broncho cephalitis	23		
Paratyphoid fever	22		

**Figure 2:**
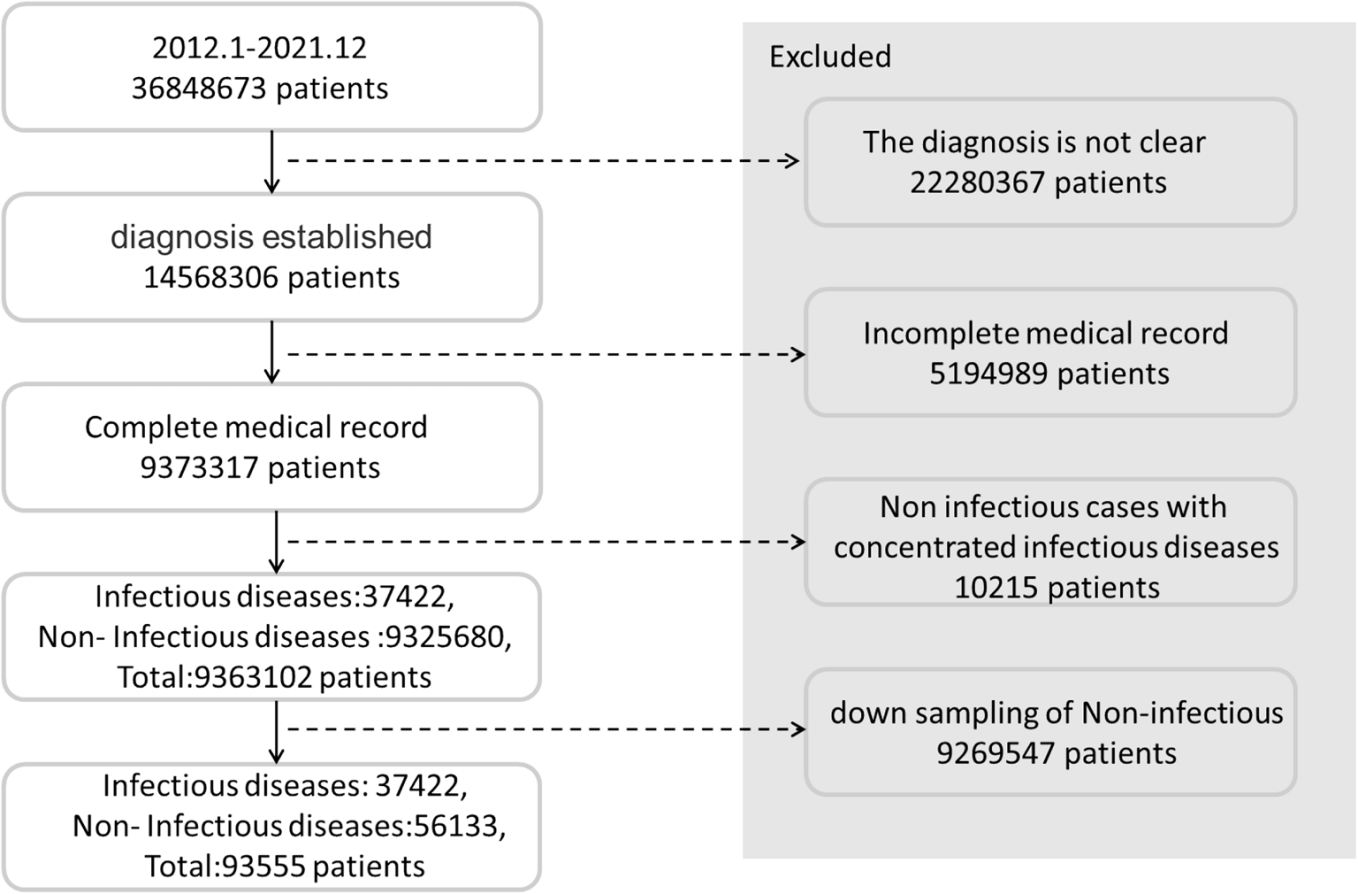
Flowchart of enrollment.

Owing to the large difference in the number of infectious and non-infectious diseases, stratified sampling was conducted. Most infectious diseases belong to obvious categories (e.g., most of them are respiratory, skin, or digestive diseases), while a few of them are related to orthopedics. Therefore, stratified sampling was carried out based on the departments from which the samples were obtained. For example, if pulmonary tuberculosis belongs to the respiratory department, then the other diseases identified in the data obtained from the respiratory department, such as pneumonia, lung cancer, chronic obstructive pulmonary disease, and bronchiectasis, are randomly selected. In addition to restricting the selection of medical records on non-infectious diseases obtained from the departments where the infectious diseases are evident, we also conducted random down-sampling of the non-infectious diseases. This step enabled the inclusion of a relatively small number of medical records on non-infectious diseases, and the number of non-infectious-disease medical records used as the training samples was 1.5 times that of the infectious cases. After the stratified and random down-sampling, 56,133 cases of non-infectious diseases were finally included. The screening process of the patients’ medical records is illustrated in Fig. 2.

To ensure maximum utilization of the information on important factors related to infectious diseases, the data used in the training model include basic patient information, time information, spatial information, patient medical records, and so on as presented in Table 2. Notably, to prevent information leakage during model training, we eliminated the test items that can clearly indicate the type of infectious disease in the test report, e.g., the new coronavirus nucleic acid test items in the medical records of COVID-19 patients. The diagnosis was used as the label of the sample; the work and home addresses were used as the key information to trace the address of the infectious disease patient; and the remaining extracted fields were used as the input features of the subsequent diagnosis model.

**Table 2:** Specific medical data.

Category	Specific data
Patient information	Gender, age
Time data	Visit time, symptom duration
Spatial data	Home address, work address
Admission records or outpatient records	Chief complaint, current medical history, history, social history, auxiliary examination
Physical examination	Temperature, blood pressure, pulse, respiration
Diagnosis	Diagnostic name, ICD-10 code
Inspection report	Inspection sub item name, result value, normal range
Radiological examination report	Radiological examination report

### Data preprocessing

#### Data extraction

Electronic medical records mainly include data in both structured and unstructured (free text) formats. The patient's basic information, diagnosis, outpatient diagnosis, and test report form the structured data of the electronic medical record, while the admission record, outpatient medical record, and examination report become the unstructured data.

Structured data directly extracts the value of the corresponding field from the electronic medical record. Specifically, information such as age, age unit, visit time, home address, and work address are extracted from the front page of the medical record; information such as the patient's diagnosis name and code are extracted from the home page or outpatient diagnosis; inspection time, inspection value, inspection scope, and other information are obtained from the report page. Then, abnormal value processing, number normalization, and standardization are carried out for continuous variables; for example, the abnormal value processing of age will filter out the ones that deviate from the normal age range. Then, according to the different age units (year, month, week, etc.), the age value is uniformly converted into the age unit; each inspection result in the inspection report is classified as “high,” “low,” or “normal” according to their normal value range. In addition, the extracted discrete variables such as diagnosis name and inspection sub-item name are unified into standard names according to the mapped relationship between the aliases and standard names in the knowledge base. Finally, the processed field data are sorted into a text sequence.

**Figure 3:**
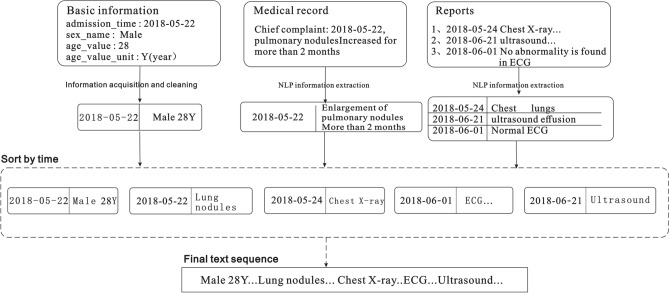
Extraction and serialization of information from electronic medical records.

For unstructured data, it is first necessary to extract information such as the chief complaint of the admission record, inspection conclusions, and inspection findings in the inspection report ^27,28^. In this case, we extract various entities (such as time, symptoms, diseases, signs, drugs, inspections, etc.), and then standardize each entity based on the British Medical Journal’s Best Practices ^29^ knowledge base, and finally obtain a data sequence. Subsequently, all the data sequences extracted from the patient's medical record are spliced according to the order of the document and inspection times to finally form the patient's input sequence for the model. The text merging process is illustrated in Fig. 3.

#### Data vectorization

To use the above-mentioned serialized data to train the model, it is also necessary to vectorize the sequence text and convert each word in the sequence into a "computable" and "structured" vector before including the data into the model as input. Word2vec, a relatively common tool for training word vectors ^30^, can transform words into dense, low-dimensional real value vectors, which capture useful syntax and contextual semantics ^31^. Therefore, we used the Skip gram in word2vec to train the word vector model. Before training the word vector, each word in the input sequence is assigned a unique identifier to identify the word. This identification number is based on the sequence of word frequency in the corpus, and the word is labelled with the highest frequency; for example, the word "male" is assigned a unique identification number < 360 > . In this study, the typical numerical values in the medical records were spliced into a new word by connecting the names, values, and units, and then a unique identifier was assigned to the spliced output; for example, "alkaline phosphatase 102 U/L" is assigned a unique identifier 891. Therefore, the Chinese word sequence is converted into a word index sequence. Then, the word vector model is trained based on the obtained word index sequence. When training the word vector model, we set the word vector dimension to 100, and a window size of 5 is adopted. In addition, considering that the time complexity of the Skip gram is expressed as window size × thesaurus size, a large number of words in the training set increase the computation time. Therefore, we set min_ count = 2 in the model, and ignore the low-frequency words that only appear once or twice.

**Figure 4:**
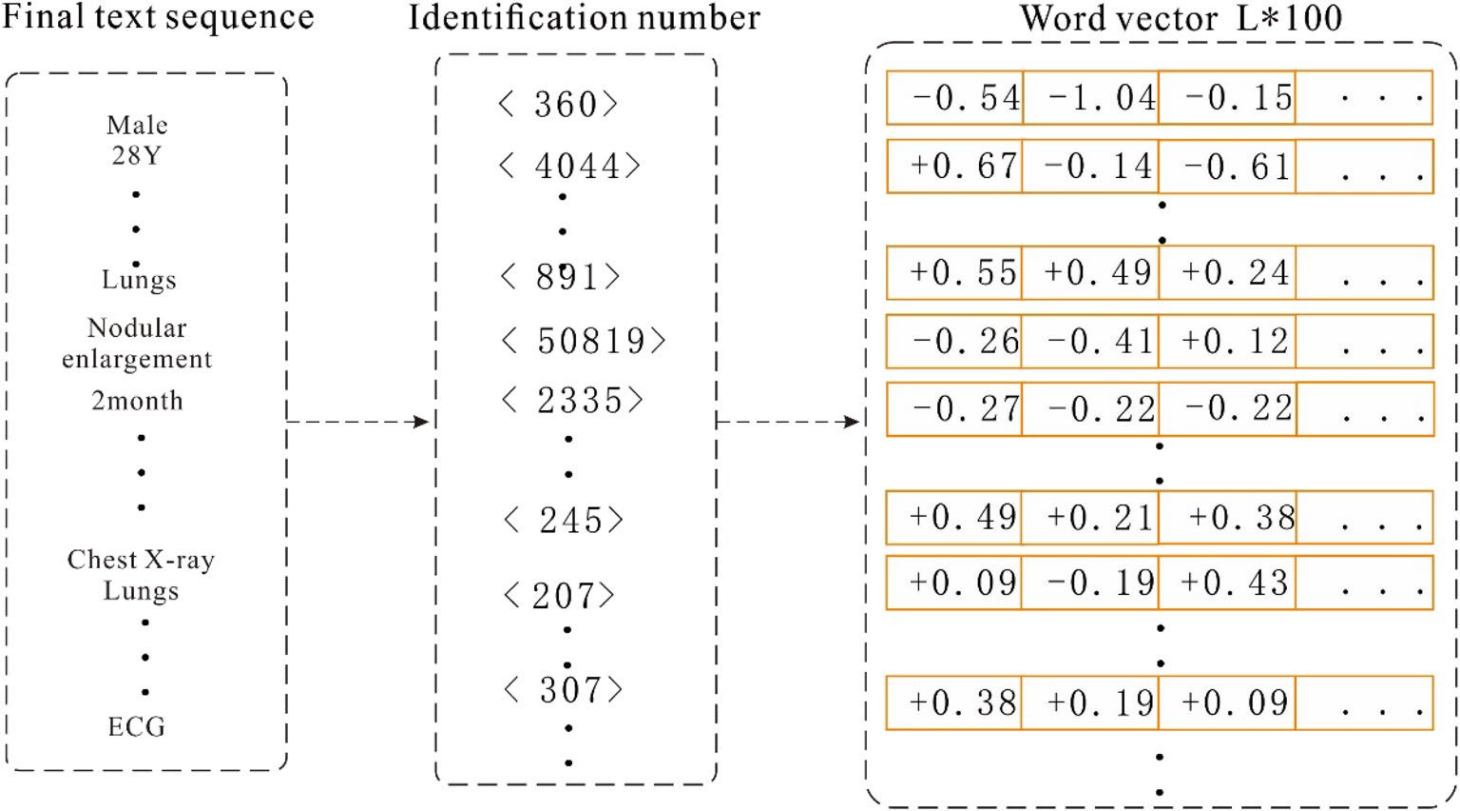
Word vector conversion.

When vectorizing sequence data, we first calculate the length of all the input sequences and denote the length L of its 98% quantile as the final length of each sequence data. Then, each input sequence is truncated and filled with 0. Next, the unique identifier corresponding to each word in the sequence is converted into a word vector representation according to the trained word vector object. Finally, all the word vectors are spliced together to form an input vector of length L × 100. The process of converting medical records into a vector is depicted in Fig. 4.

### Unbalanced dataset processing

To alleviate the impact of data imbalance on the model results, down sampling was performed in the first layer model in this study. The number of medical records on non-infectious diseases is 1.5 times of that on infectious diseases. In the training of the two-layer model, we added category weight processing to provide categories with fewer samples a higher calculation weight, expressed by formula (1), and use them extensively for model training:

1 $${w}_{k}=\frac{{N}_{all}}{C\cdot {N}_{k}}$$

where ${w}_{k}$ represents the weight of the $k$ class, ${N}_{all}$ represents the total number of samples in the dataset, $C$ denotes the total number of categories ($C$ = 8), and ${N}_{k}$ represents the number of samples of the $k$ class. When the weight is not changed, the weight for each category is represented by the average attention degree ($1/C$), and the weight calculation formula satisfies:

Category weight × proportion of the number of category samples in the total dataset = Average attention.

### Proposed EIDDM architecture

**Figure 5:**
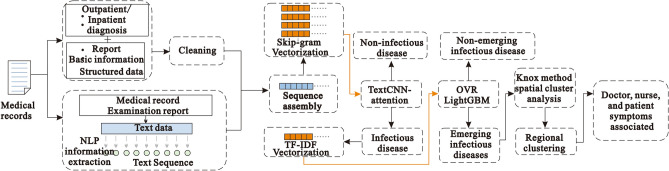
EIDDM flow.

In this study, we mainly used the EIDDM (TextCNN-Attention + LightGBM), a regional clustering analysis, and symptom association of medical staff to predict the outbreak of emerging infectious diseases. Although no current case of emerging infectious diseases such as SARS, COVID-19, monkeypox, etc., was analyzed in this study, their performance is similar to that of the known infectious diseases; further, their characteristics are similar to those of regional clusters of infectious diseases. Therefore, we used a two-layer model to predict the number of people infected with suspected emerging infectious diseases, and then used their contact addresses to evaluate the possibility of existence of regional clusters. The EIDDM flow is shown in Fig. 5. Finally, through the symptom association analysis of medical staff, we assessed the probability of "human-to-human transmission.". The results of the analysis of interpersonal association transmission ^32–34^ are expected to promote further follow-up epidemiological studies on transmission routes, gene sequencing, and prevention and control programs.

#### First layer of model with TextCNN-attention

Because of the large amount of medical record data and high-dimensional feature sequence text data in the first layer model used in this study, the model selects the text-based convolutional neural network TextCNN for classification. This network considers both convolution extraction and timing. The word embedding features of the long text sequences are composed of admission records, and thus, the inspection and testing documents have different effects on the classification results. The features that have a greater impact on the model decision are given a greater weight of attention via the Multi-Head Attention mechanism, and then the key and interference features of the text can be distinguished. Therefore, the introduction of the Attention mechanism in TextCNN can effectively enhance the feature extraction capability of the model.

**Figure 6:**
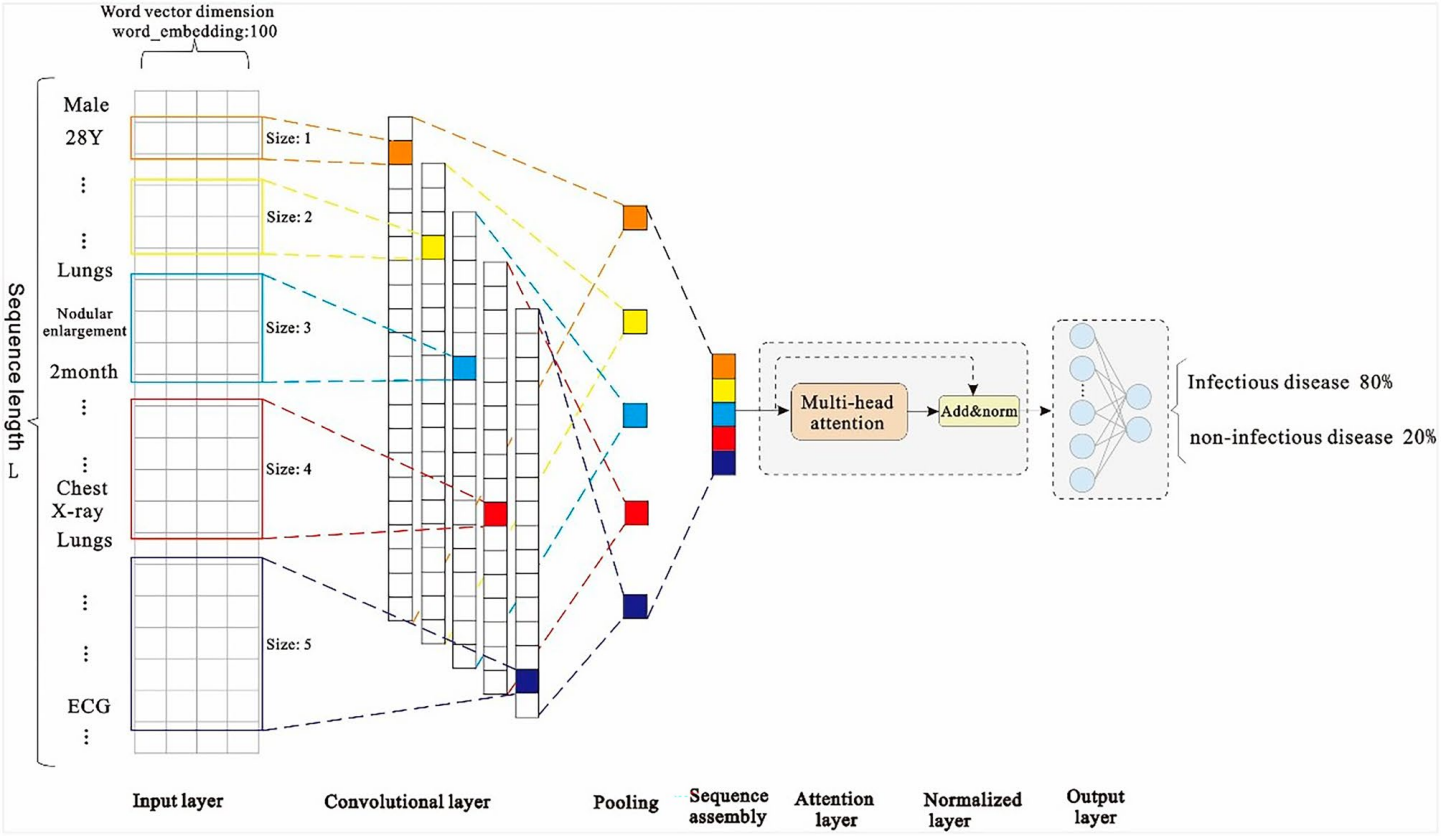
First layer of the EIDDM-TextCNN-Attention model.

First, zero-padding is used for the filling to ensure that the length of the input feature sequence is consistent in the model. The final maximum sequence length is L (L = 5061), indicating that the input sequence size is 5061 (after the operation: word vector layer × 100). After the word embedding layer, a word embedding matrix with a size of 5061 × 100 is formed. The parameters of the word embedding layer are initialized using the Skip-gram method. Notably, one-dimensional convolution is used in this convolutional neural network. We consider the number of samples, hardware equipment performance, model complexity, case data characteristics, and other factors, and use the grid search ^35^ method to set multiple values for the same parameter in different value domains and magnitude ranges in descending order. By comparing the accuracy of the trained model using the test set, five filters with dimensions of 1 × 100, 2 × 100, 3 × 100, 4 × 100, and 5 × 100 are set. After the convolution layer, five convolution expressions, L × 100, (L-1) × 100, (L-2) × 100, (L-3) × 100, and (L-4) × 100 are obtained, respectively. Then, we add the max pooling layer to reduce the dimensions of the filter-layer features and splice the pooled vectors; the spliced dimensions are 500. The reduced-dimension spliced pooled vectors are input into the Muti-Head Attention layer with multi-head parameter = 8 and head_dim parameter = 16. Then, we connect a residual (size: 500) with the normalization layer. Finally, the vector is expanded, and in order to prevent overfitting, the dropout loss mechanism is used as the input of the sigmoid layer. The structure of the first layer of the EIDDM is shown in Fig. 6.

#### Second layer of EIDDM with LightGBM

Should the first layer of evaluation indicate the presence of an infectious disease, the medical record text sequence is then transformed into Term Frequency-Inverse Document Frequency (TF-IDF) values, which are subsequently inputted into a secondary model for assessing whether the sequence constitutes an emerging infectious disease. TF-IDF serves as a textual vectorization representation method. Term Frequency (TF) computes the frequency of occurrence of a specific term within the current medical record and normalization. Inverse Document Frequency (IDF) is used to ascertain whether a term is prevalent across multiple medical records. If a term appears frequently in numerous medical records, it is likely to be a common term with lower discriminatory power across the entire corpus of medical records. TF-IDF is the product of multiplying TF and IDF. It combines the importance of each term within the current medical record with its significance in the overall corpus of medical records. For terms that appear frequently in the current medical record but infrequently in the entire corpus, the TF-IDF value is higher, emphasizing their significance within the current medical record. Through TF-IDF transformation, the input text sequence is represented as a vector.

Multi-classification algorithms such as random forest, XGBoost, and LightGBM are decision tree-based ensemble classifiers, and as a result, their prediction accuracies are higher than that of a single decision tree model. The efficiency and scalability of XGBoost and random forest are not ideal for data with a large sample size and high feature latitude, because during the splitting of the tree nodes, they need to scan the eigenvalues of each feature to locate the optimal cut points, which is a time-consuming process. To solve the above problems, LightGBM proposed gradient-based one side sampling and exclusive feature bundling (EFB) to sample and reduce the feature dimensions, respectively. LightGBM can focus on the "not fully trained" sample data during the model training. Simultaneously, a histogram algorithm is used to accelerate the search for segmentation points, and large-scale data are input into the histogram; this step reduces the feature memory size and accelerates the model training.

In addition, for category features, on-hot coding is employed by gradient boosting decision tree GBDT, XGBoost, and random forest. However, one-hot coding is easy to overfit, and the tree is relatively deep to achieve better results. LightGBM uses the EFB algorithm to optimize the support of the category features. Through the combination of sparse features and binding of mutually exclusive features, the category features can be directly input without any additional 0/1 expansion, and this process further optimizes the training speed of the model. Therefore, considering the time, memory, accuracy, and other aspects, LightGBM with a fast iteration speed and strong interpretability is selected as the second layer of the classification model used in this study, and the prediction performances of random forest and XGBoost are compared.

The objective of this study is to identify emerging infectious diseases, where "emerging" indicates that the disease category was not present in the current training dataset. In order to perform this identification, the study employs the One vs. Rest strategy ^36^. For the N categories present in the training dataset, N LightGBM models are trained, with each model undertaking a binary classification task. During the prediction phase, an initial threshold is established. Novel test samples undergo probability calculations through individual binary classifiers. If the computed probability surpasses the threshold, it indicates membership in a singular category; conversely, probabilities below the threshold suggest membership in the remaining categories. In cases where the predictive probabilities from all classifiers are below the threshold, indicating that all N models collectively classify the test sample into non-target categories—meaning the sample is dissimilar to any of the infectious disease categories currently included in the models—it is inferred that the sample pertains to an emerging infectious disease. We substantiate the validity of the One vs. Rest approach for recognizing novel categories by using a spatial ensemble methodology. Specifically, assume that the current training set contains three categories, A/B/C, and the test sample X. Then, three dichotomous OvR experiments are conducted: (1) A / R(BC), (2) B / R(AC), (3) C / R(AB). Finally, three models, viz. Model-1 (M1), Model-2 (M2), and Model-3 (M3), are obtained through the training. For the test sample X, the probability P (A | M1) that X belongs to category A, the probability P (B | M2) that X belongs to category B, and the probability P (C | M3) that X belongs to category C are obtained using the model. Finally, X is judged based on the set threshold and results of the three models.

**Figure 7:**
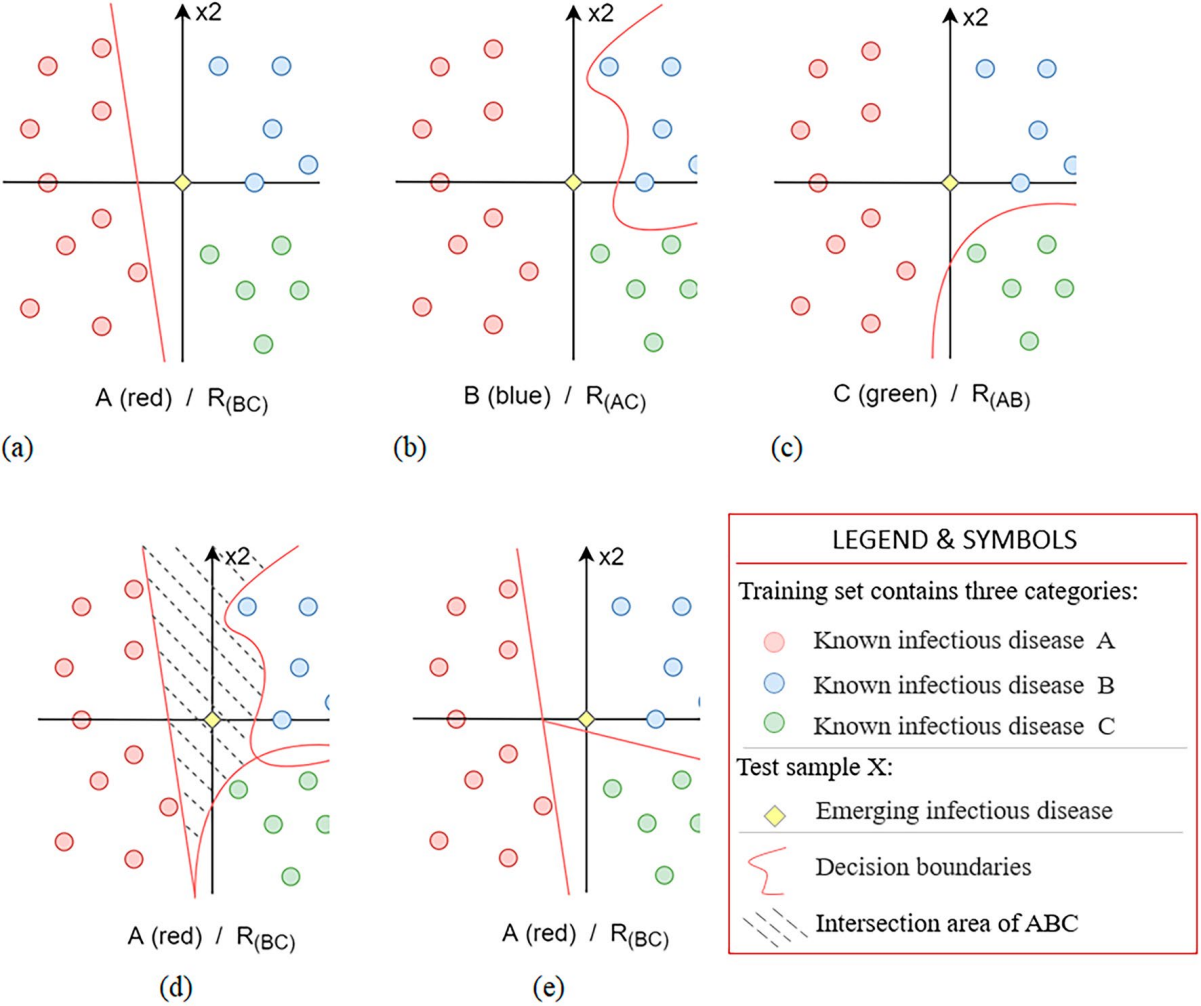
OvR strategy-based identification of test results for new categories (illustrated in a two-dimensional space).

For the above-mentioned process, the following condition is assumed:

2 $${(\mathrm{BC})}_{\mathrm{M}1}\cap {(\mathrm{AC})}_{\mathrm{M}2}\cap {(\mathrm{AB})}_{\mathrm{M}3}\ne \mathrm{\varnothing }$$

where ${(\mathrm{BC})}_{\mathrm{M}1}$ represents the space area of category R(BC) divided by the decision boundary of M1 in the current feature space. The division methods of ${(\mathrm{AC})}_{\mathrm{M}2}$ and ${(\mathrm{AB})}_{\mathrm{M}3}$ follow a similar process. Figure 7 (a), (b), and (c) represent the test results of the three models (shown in a two-dimensional space), respectively. The red line represents the decision boundary obtained from the model training, and the yellow point represents the feature space of the test sample X. When X is predicted as category R (BC), R (AC), and R (AB) by each model, it is judged as a new category. As shown in Fig. 7 (d), when sample X is located in the intersection area of the three R categories, it represents a new category that does not belong to A/B/C. However, when the decision boundary of each model completely cuts the feature space (i.e., there is no intersection area in the R category space of each model, as shown in Fig. 7 (e)), the sample X must belong to one of the A/B/C categories, and thus, it cannot be labeled as a new category.

In summary, the identification of new categories using the OvR strategy depends on the condition that an intersection exists between the R category regions of each model; that is, formula (2) must be satisfied. In the current experiment, the TF-IDF data feature is a high-dimensional space of 84,411 dimensions, and the probability that the decision boundaries of each model partially overlap or are perfectly cut is extremely small. Therefore, these experimental results satisfy the assumption of formula (2), and the OvR strategy-based new category identification method can be implemented in this model.

#### Regional cluster analysis

Using the EIDDM, we can determine the probability of a single case sample being an emerging infectious disease. The time series data obtained from the simultaneous spatiotemporal monitoring of infectious diseases are integrated into the complete patient record in the two-layer model, which meets the requirements of time monitoring. We also need to consider the spatial cluster analysis of the family and work addresses in the patient record to reduce the occurrences of positive false results for single case prediction in real applications of this model in medical institutions. In epidemiology, clustering of infectious diseases is a common phenomenon. Presently, statistical methods, including flexible spatial scanning statistical analysis ^37^, Rogerson spatial pattern-based analysis method ^38^, Knox method that uses only the spatiotemporal information of the cases ^39^, and so on, are widely employed for the detection of spatial clusters of infectious diseases. The flexible, Rogerson, and Turnbull methods first divide the whole research area into multiple sub-regions for the analyses and require population data of each sub-region, including the total population and migration data. In practice, it is difficult to select the most appropriate sub-region division method for the study area; therefore, the above three methods are not applicable in this study. The Knox method does not need to divide the whole region into molecular regions; moreover, it does not require demographic data of sub-regions as input parameters. Instead, this method only requires the case information and spatiotemporal data without control group and susceptible population data ^40^. The Knox method is a global test method for evaluating spatiotemporal aggregation and analyzes the location and time of the disease onset. The test statistic $X$ is the number of case pairs that are close in space and time and is represented by formula (3); its expected formula is expressed as formula (4).

3 $$X\left(s,t\right)=\sum_{I=1}^{N}\sum_{j=1}^{i-1}{a}_{ij}^{s}{a}_{ij}^{t}$$

4 $$E\left[X|{N}_{S},{N}_{t}\right]=\frac{{N}_{s}{N}_{t}}{N}$$

where *N* is the number of cases; ${a}_{ij}^{s}$= 1 if the cases *i* and *j* are similar in space, and ${a}_{ij}^{s}$ = 0 if the cases are dissimilar; ${a}_{ij}^{t}$ = 1 if the cases *i* and *j* are similar in time, and ${a}_{ij}^{t}$ = 0 if the cases are dissimilar; and *s* and *t* represent the prespecified space and time distances, respectively. If the difference between *X* and its expected *E* is statistically significant, then there exists a spatiotemporal clustering feature. We calculate the onset date and case space distances of the case pair. If these distances are less than the prespecified time and space threshold, then a "near" case is obtained. The prespecified time threshold is the incubation time of each infectious disease, and the prespecified space threshold is 200, 500, 1000, 2000 m, and other different intervals. We count the number of cases with "near" statistical time ${N}_{t}$ as well as those with "near" distance ${N}_{s}$.

When acquiring the spatial data, we first collect the detailed contact address, home address, work address, and other information of the people suspected to be infected with emerging infectious diseases from the patient's personal information. Second, we the longitude and latitude of each address of the patient as the coordinate datapoint of the patient from the China Gaode Map Service ^41^; for example, the longitude and latitude of the contact the address "Peking University, No. 5, Yiheyuan Road, Haidian District, Beijing" is (116.310905,39.992806), which was obtained from the map of Gaode.

Most early studies on the statistical Knox method were focused on extracting approximate values using methods such as Poisson distribution, Barton–David method, Monte Carlo method, and so on. However, in this study, the Monte Carlo method was used for analyzing the test statistics $X$ ^42^. We performed several random simulations, and in each simulation, the $X$ cases are randomly marked as *N* occurrences. Further, the reference distribution is obtained by calculating the test statistics of each random number. The specific steps of this method are as follows:

Calculate the actual statistics $X$ of the original dataset;

The spatial distance between case pairs is fixed, whereas the time distance is rearranged to generate a new random dataset;

Recalculate the statistics for randomly rearranged datasets;

The calculation was repeated 999 times for each infectious disease, and 999 new statistics were obtained;

Count the number of each new statistic and arrange it in ascending order according to the size of the new statistic;

Estimate of $P$-value as shown in formula (5).

5 $$P=\frac{k}{N+1}$$

where $k$ ≥ $X$ in the new statistics, and $N$ is the total number of new statistics.

Finally, the $P$-value is compared with the threshold of 0.05. If $X$ > $E$, then we obtain $P$-value < 0.05, which indicates that the case has spatiotemporal aggregation.

#### Early warning of doctor, nurse, and patient association symptoms

For the case when the classification model predicts the onset of an emerging infectious disease, an algorithm for symptom related monitoring of medical personnel is proposed to aid in hospital infection management and epidemic prevention and control. First, we use formula (6) to identify the occurrence of an infection among the medical staff, and then use formula (7) to determine whether the infection value exceeds the expected infection value. If it exceeds the expected infection value, then we focus on monitoring the current target patients and medical staff receiving treatment, so that public health experts can intervene to assess the possibility of "human-to-human" transmission of emerging infectious diseases. According to the initial time point, contact times, and contact status of the target patients and their medical staff, the occurrence of the same symptoms in doctors and nurses is deduced within seven days after receiving the treatment to enable an epidemiological analysis.

Initially, the symptom sets of patients with Emerging Infectious Diseases predicted by the hierarchical diagnostic model, were categorized as H = {${h}_{1},{h}_{2}$…${h}_{j}$}. For the primary care physicians of the target patients, a symptom vector V and a set of symptom vector values {${v}_{1}$,${v}_{2}\dots {v}_{j}$}were created using data from the patients' medical records at the time of first contact. For the secondary contact connection between the initial contact medical staff and the remainder of the medical staff in the same department, the symptom vector U and symptom value vector set {${u}_{1}$,${u}_{2}\dots {u}_{j}$}were built. We calculated the highest frequency symptom using term frequency–inverse document frequency (TF-IDF) for the sets H, V and U, and recorded it as ${a}_{1}.$ The symptoms with the highest frequency ${r}_{i}$ were recorded as prominent symptoms, and other symptoms were recorded as concomitant symptoms. The reference symptom vector K and the corresponding symptom value vector set{${k}_{1},{k}_{2}\dots {k}_{\mathrm{j}}$} of the emerging infectious diseases are set according to the clinical observations. Zhu et al. ^43^ used the Pearson correlation coefficient to measure the correlation between the symptoms and calculate the difference relationship between the target patient H and the medical staff ($U\cup V$) as well as preset the reference symptom K. In this case, the Pearson correlation coefficient $1-|p|$ is the difference factor $\partial$. If this is not the case, then the difference factor $\partial$ is 1, indicating that the symptoms are not similar. If all the difference factors $\partial$ satisfy the condition $1>\ge 0$, then the symptoms are similar (${n}_{1}$ represents the total number of similar symptoms; ${n}_{2}$ represents the total number of non-similar symptoms of the target patient ${h}_{i}$; and ${n}_{3}$ represents the total number of non-similar symptoms of the corresponding medical staff).

Formula (6) is used to calculate the infection value $Y$, which includes the calculations of both similar and dissimilar symptoms. Experts can determine whether to include the dissimilar symptoms in the calculation of the infection value to set the evaluation parameters $\varepsilon$. If the dissimilar symptoms are included, then the value is 1; otherwise, 0.

6 $$ Y = 1 - \sum\limits_{i = 1}^{n1} {\left( {s_{i0} - s_{i1} } \right)^{2} + \left( {\sum\limits_{j1 = 1}^{n2} {s_{j0} \varepsilon_{j0} - \sum\limits_{j2 = 1}^{n3} {s_{j1} \varepsilon_{j1} } } } \right)}^{2} \times \ln \left( {1 + \sum \partial } \right) $$

where $s_{i0}$ represents the vector value of the ${i}_{th}$ similar symptom based on the target patient; $s_{i1}$ represents the vector value of the ${i}_{th}$ similar symptom based on the medical staff; $s_{j0}$ represents the vector value of the symptom based on the ${j}_{th}$ dissimilar symptom index of the target patient; $\varepsilon_{j0}$ represents the evaluation coefficient based on whether the ${j}_{th}$ dissimilar symptom index of the target patient has a reference value; $s_{j1}$ represents the vector value of the symptom based on the ${j}_{th}$ dissimilar symptom index of the medical staff; $\varepsilon_{j1}$ represents the evaluation coefficient based on whether the ${j}_{th}$ dissimilar symptom index of the medical staff has a reference value; and $\sum \partial$ represents the cumulative sum of the difference factors corresponding to each prominent symptom.

The determined infected value indicates the possibility of an associated infection risk between the patient and the medical staff. Further, calculating the deterioration probability P metric of the corresponding medical staff according to certain prominent symptoms of the staff is essential to assess the current risk, reduce the large amount of interference monitoring caused by more common symptoms, and focus on the medical and nursing population beyond the research and judgment of public health experts. The calculation formula of deterioration probability $P$ is expressed as (7):

7 $$ P = \frac{Y}{{Y_{1} }} \times {\text{exp}}\left( {\frac{{\sum R \times \frac{{\left| {Y - Y_{0} } \right|}}{{\left| {Y_{0} - Y_{1} } \right|}}}}{m1} - \Im } \right) $$

where $Y_{0}$ represents the left boundary value of the infection level corresponding to $Y$; $Y_{1}$ represents the right boundary value of the infection level corresponding to *Y* and $Y_{0}$ is less than $Y_{1}$. The boundary values $Y_{0}$ and $Y_{1}$ are determined by experts according to historical conditions. The parameter $\sum R$ is the total influence factor of all the corresponding prominent symptoms that deteriorate the health of the patient, and the value is determined based on the prominent symptoms ${r}_{i}$ determined by the experts; typically, the value is in the range of ($\mathrm{0,1})$. Further, in formula (7), $m1$ represents the total number of corresponding prominent symptoms; and $\Im$ indicates the adjustment parameter when $\left| {Y - Y_{0} } \right| \le \left| {Y - Y_{1} } \right|$ ($\Im = 0.2$ for $\left| {Y - Y_{0} } \right| \le \left| {Y - Y_{1} } \right|$; otherwise, $\Im$ = $\{\mathrm{0,1}\}$). We compare the results of hierarchical monitoring according to the deterioration probability $P$ and preset probability ${P}_{0}$. Evidently, if *P* > *P*_*0*_, then we should focus on monitoring the corresponding medical staff. In the opposite case, we should conduct a routine monitoring of the corresponding medical staff.

### Performance evaluation

Given the absence of authentic test medical records for emerging infectious diseases in practical settings, this study hypothetically designated various known infectious diseases as emerging infectious diseases and excluded them from the training dataset during model construction. Subsequently, these hypothetical medical records for the designated infectious diseases were used as test cases for emerging infectious diseases in the well-trained model in order to evaluate the model's predictive capabilities. The prediction process of the EIDDM model comprises two stages: first, the first layer model is employed to determine if the case is an infectious disease; if it is, then the secondary layer model is invoked to determine whether it is an emerging infectious disease or not. As a result, it is essential to assess the accuracy of the first layer model in predicting infectious diseases and the accuracy of the secondary layer model in predicting emerging infectious diseases separately. The evaluation method of the first layer model is presented in Table 3.

**Table 3:** Confusion matrix of the first layer model.

	Infectious diseases	Non-infectious diseases
Predicted infectious disease	$T{P}_{1k}$	$F{P}_{1k}$
Predicted non-infectious disease	$F{N}_{1k}$	$T{N}_{1k}$

In Table 3, $$k$$ indicates that the K^th^ infectious disease is predicted as an emerging infectious disease; $$T{P}_{1k}$$ indicates that the real medical record is an infectious disease, and the model correctly predicts the number of samples for the infectious disease; $$F{P}_{1k}$$ indicates that the real medical record is a non-infectious disease, and the model incorrectly predicts the number of samples for the infectious diseases; $$F{N}_{1k}$$ indicates that the real medical record is an infectious disease, and the model incorrectly predicts the number of samples as non-infectious disease; and $$T{N}_{1k}$$ indicates that the real medical record is a non-infectious disease, and the model correctly predicts the number of samples for the non-infectious diseases. The accuracy, sensitivity ($$T{PR}_{1k})$$, and $${FNR}_{1k}$$ of the first layer model prediction are evaluated using formulae (8)–(10), respectively:

8 $${Accuracy}_{1k}=\frac{T{P}_{1k}+T{N}_{1k}}{T{P}_{1k}+F{P}_{1k}+F{N}_{1k}+T{N}_{1k}}$$

9 $${Sensitivity}_{1k}=T{PR}_{1k}=\frac{T{P}_{1k}}{T{P}_{1k}+F{N}_{1k}}$$

10 $${FNR}_{1k}=1-T{PR}_{1k}=\frac{{FN}_{1k}}{T{P}_{1k}+F{N}_{1k}}$$

The proportion of non-infectious diseases in the medical records correctly predicted as non-infectious diseases is referred to as specificity (${Specificity}_{1k}$) or true negative rate ($T{NR}_{1k}$). Conversely, the rate at which non-infectious diseases are erroneously predicted as infectious is known as the false positive rate. The formulae for calculating specificity and false positive rate are as follows:

11 $${Specificity}_{1k}=T{NR}_{1k}=\frac{T{N}_{1k}}{T{N}_{1k}+F{P}_{1k}}$$

12 $${FPR}_{1k}=1-T{NR}_{1k}=\frac{{FP}_{1k}}{T{N}_{1k}+F{P}_{1k}}$$

Furthermore, it is necessary to assess how many of the diseases identified by the model as infectious diseases are truly infectious diseases, which is known as the positive predictive value (${PPV}_{1k}$). Its calculation formula is as follows:

13 $${PPV}_{1k}=\frac{T{P}_{1k}}{T{P}_{1k}+F{P}_{1k}}$$

Here, "$T{P}_{1k}+F{P}_{1k}$" represents the total number of medical cases predicted as infectious diseases in the first layer.

In the set of model predictions for non-infectious diseases, it is essential to determine how many are truly non-infectious diseases, referred to as the negative predictive value (${NPV}_{1k}$). Its calculation formula is as follows:

14 $${NPV}_{1k}=\frac{T{N}_{1k}}{T{N}_{1k}+F{N}_{1k}}$$

Here, "$T{N}_{1k}+F{N}_{1k}$" represents the total number of medical cases predicted as non-infectious diseases in the first layer.

Among the subset of cases predicted as infectious diseases by the first layer (${TP}_{1K}$ and ${FP}_{1K}$), the second layer model is subsequently employed to predict whether they are emerging infectious diseases. Assume that for true medical records classified as emerging infectious diseases, the second layer model correctly predicts $T{UK}_{2k}$ samples as emerging infectious diseases and incorrectly predicts $F{K}_{2k}$ samples as non-emerging infectious diseases. For true medical records classified as non-infectious diseases, the secondary layer model incorrectly predicts $F{UK}_{2k}$ samples as emerging infectious diseases.

The sensitivity of the second layer model for the K^th^ emerging infectious disease, denoted as Sensitivity_2k_ (also referred to as True Positive Rate), represents the proportion of correctly predicted emerging infectious disease cases in the medical records of this emerging infectious disease. Conversely, the false negative rate ($F{NR}_{2k}$) represents the proportion of cases from this infectious disease category that was incorrectly predicted as non-emerging infectious diseases. The formulas for calculating sensitivity and $F{NR}_{2k}$ are as follows:

15 $$ S{\text{ensitivity}}_{2k} = TPR_{2K} = \frac{{TUK_{2k} }}{{TUK_{2k} + FK_{2k} }} $$

16 $$ FNR_{{2{\text{k}}}} = 1 - TPR_{2k} = \frac{{FK_{2k} }}{{TUK_{2k} + FK_{2k} }} $$

Here, ${TUK}_{2k}$ + ${FK}_{2k}$ = ${TP}_{1k}$ represents the total number of medical records for the ${K}_{th}$ emerging infectious disease that were correctly predicted as infectious diseases by the first layer.

In the second layer model, true medical records categorized as non-infectious diseases are incorrectly predicted as infectious diseases. Hence, the specificity is 0, and ${NPV}_{2k}$ is also 0. The false positive rate is divided into two components, with one being predicted as emerging infectious diseases and the other as non-emerging infectious diseases. The false positive rate for being predicted as emerging infectious diseases is calculated as given in Formula (17).

17 $$ FPR{}_{{2{\text{k}}}} = \frac{{FUK_{2k} }}{{FUK_{2k} + FK2_{2k} }} $$

Here, ${FUK}_{2k}$ + ${FK}_{2k}$  = ${FP}_{1k}$, representing the total number of medical records for non-infectious diseases that were incorrectly predicted as infectious diseases by the first layer.

EIDDM combines the overall accuracy of the two layers, denoted as ${A\mathrm{ccuracy}}_{k}$, which represents the ratio of correctly predicted medical records for emerging infectious diseases and non-infectious diseases to the total number of test medical records. Specifically, the number of correctly predicted medical records for emerging infectious diseases is ${TUK}_{2k}$, while the number of correctly predicted medical records for non-infectious diseases is ${TN}_{1k}$, which is the count of cases correctly predicted as non-infectious diseases by the first layer. Therefore, the formula for calculating the overall model accuracy is as follows.

18 $$ A{\text{ccuracy}}_{k} = \frac{{TUK_{2k} + TN_{1k} }}{{TP_{1k} + FP_{{1{\text{k}}}} + FN_{1k} + TN_{1k} }} $$

By considering the first layer as well as secondary prediction models, we can derive ${Sensitivity}_{k}$, also known as true positive rate (${TPR}_{k}$), for the K^th^ infectious disease. TPR represents the proportion of correctly predicted emerging infectious disease cases within the medical records of that specific infectious disease. Conversely, the false negative rate (${FNR}_{k}$), which is the rate of cases from the emerging infectious disease category being incorrectly predicted as non-infectious diseases or known infectious diseases, is calculated as given below:

19 $$ S{\text{ensi}}tivity_{k} = TPR_{k} = \frac{{TUK_{2k} }}{{TP_{1k} + FN_{1k} }} $$

20 $$ FNR_{K} = 1 - TPR_{K} = \frac{{FN_{1k} }}{{TP_{1k} + FN_{1k} }} + \frac{{FK_{2K} }}{{TP_{1K} + FN_{{1{\text{k}}}} }} $$

As non-infectious diseases are predicted as non-infectious diseases only in the first layer model, whereas they may be predicted as either emerging infectious diseases or non-emerging infectious diseases in the secondary layer, the proportion of correctly predicted non-infectious disease cases within all non-infectious disease medical records, referred to as ${Specificity}_{k}$ (also known as true negative rate, ${TNR}_{k}$), remains identical to the Specificity of the first layer, denoted as ${Specificity}_{1k}$.

Conversely, within the set of cases predicted as non-infectious diseases, the true rate of non-infectious diseases, known as negative predictive value (${NPV}_{k}$), is also equivalent to the NPV of the first layer model, denoted as ${NPV}_{1k}$.

For the EIDDM model's predictions of emerging infectious diseases, the positive predictive value for true emerging infectious disease cases, denoted as ${PPV}_{k}$, is used as the result for the secondary layer, which is also equivalent to ${PPV}_{2k}$. The false positive rate is divided into two components, with one being predicted as emerging infectious diseases while the other as non-emerging infectious diseases. The false positive rate for cases being predicted as emerging infectious diseases is calculated as below:

21 $$ FPR_{{\text{k}}} = \frac{{FUK_{2K} }}{{TN_{1K} + FP_{1K} }} $$

## Ethical approval

The study was approved by the Medical Science Research Ethics Committee of Peking University Third Hospital (serial number: IRB00006761-M2022287). All methods were performed under the relevant guidelines and regulations. The informed consent was waived by Peking University Third Hospital.

## Results

### Model parameter setting

The super parameter setting has a great impact on the accuracy of the model. Therefore, the grid search method was adopted in this study. The parameter search method uses a large step size for a rough search. After the parameters are preliminarily determined, a small step size is used for a fine search near the parameters. The optimal parameters are searched from the value range of each parameter by iteration. In this study, 520 batches were used for training, and the learning rate was set as 0.001. We set early_stopping_rounds to prevent overfitting by the model, and the corresponding optimal parameters are shown in Table 4.

**Table 4:** TextCNN-Attention parameter setting of the first layer model.

Model	Parameters	Explanations	Range	Step length
TextCNN-	Learning rate = 0.001	Learning rate	[0.0005,0.001,0.005,0.01,0.05,0.1	
Attention	Conv1D = 5	Multiple attention	0.3–0.7	0.1
	multi_heads = 8	Dimension of each head	200–800	80
	head_dim = 16	Neuronal inactivation	5–15	5
	dropout = 0.5	Number of samples for one training		
	batch size = 520	Early stop setting		
	early_stopping_rounds = 10			

The second layer model uses LightGBM and compares the prediction performances of the XGBoost and random forest models. Each algorithm performs a grid search to optimize the model parameters. The main adjusted parameters and their optimal results are listed in Table 5.

**Table 5:** Parameter setting of the second layer model.

Model	Parameters	Explanations	Range	Step length
LightGBM	n_estimators = 150	The maximum number of iterations	100–200	20
	max depth = 8	Maximum depth of tree	5–15	1
	num leaves = 50	Number of leaves of the tree	10–100	10
	learning rate = 0.1	Learning rate	[0.01,0.1,1]	
XGBoost	n_estimators = 180,	The maximum number of iterations	100–200	20
	max depth = 6	Maximum depth of tree	5–15	1
	colsample bytree = 0.6	Proportion of features used by each tree to all features	0.5–0.8	0.1
	learning rate = 0.1	Learning rate	[0.01,0.1,1]	
Random Forest	n_estimators = 120	The maximum number of iterations	100–200	20
	max depth = 13,	Maximum depth of tree	5–20	1
	min_samples_leaf = 5	Minimum number of samples required at leaf node	1–15	2
	max features = 0.7	Number of features in the selected feature subset	0.5–0.8	0.1

### Model results

The testing process first inputs the case into the first layer model to identify whether it is an infectious disease. If it is an infectious disease, then the input is sent to the second layer model to identify whether it is an emerging infectious disease. Finally, we calculate the accuracy of predicting emerging infectious diseases and non-communicable diseases, the sensitivity of emerging infectious diseases being correctly predicted, as well as the false positive rate and false negative rate. The results of the first layer model (TextCNN-Attention) are shown in Table 6.

**Table 6:** TextCNN-Attention results for various infectious diseases.

Category of infectious diseases	Number of emerging infectious diseases for test	Number of non-infectious disease for test	$${Sensitivity}_{1k}$$ (%)	$$ {Specificity}_{1k}$$ (%)	$${PPV}_{1k}$$ (%)	$${NPV}_{1k}$$ (%)	$${Accuracy}_{1\mathrm{k}}$$ (%)
Viral hepatitis	23,929	35,894	64.90	98.11	95.82	80.74	84.83
Influenza	3890	5835	73.47	97.43	95.01	84.64	87.85
Tuberculosis	5313	7970	70.88	95.80	91.83	83.15	85.83
Hand-foot-and-mouth disease	1573	2360	85.63	98.39	97.26	91.13	93.29
Syphilis	916	1374	89.74	97.60	96.14	93.45	94.45
Infectious diarrhea	508	762	90.75	91.60	87.81	93.69	91.26
Scarlatina fever	409	614	92.67	94.95	92.44	95.11	94.04
COVID-19	87	131	86.21	96.18	93.75	91.30	92.20
Weighted average by test sample size	–	–	68.91	97.57	95.07	82.63	86.11

Based on the experimental results of the first-layer model, it can be observed that the sensitivity for predicting infectious diseases such as scarlet fever and infectious gastroenteritis exceeds 90%, while other infectious diseases can be predicted with a sensitivity of over 60%. The average specificity for non-infectious diseases is 97.57%, with an average negative predictive value (${NPV}_{1k}$) of 82.63%, indicating a low risk of misdiagnosing non-infectious diseases as infectious (false positive rate). The average positive predictive value for the eight infectious diseases (${PPV}_{1k}$) is 95.07%, demonstrating the model's ability to avoid "misdiagnoses." The overall average accuracy of the model is 86.11%. Thus, the first-layer model proves effective in predicting infectious as well as non-infectious diseases.

The cases denoted as infectious diseases by the first layer model are sent to the second layer model, which identifies whether they are non-emerging or emerging infectious diseases. The second layer model was tested using the LightGBM, XGBoost, and Random Forest algorithms, and the corresponding test results are presented in Table 7.

**Table 7:** Second layer model (LightGBM and other models) test results for different diseases (threshold: 0.5).

Category of infectious diseases	The number of infectious diseases predicted by the first-level model(TP_1K_ + FP_1k_)	Sensitivity_2k_			FNR_2k_			FPR_2k_		
		LGBM (%)	XGBoost (%)	RF (%)	LGBM (%)	XGBoost (%)	RF (%)	LGBM (%)	XGBoost (%)	RF (%)
Viral hepatitis	16,207	90.59	95.73	89.04	9.41	4.27	10.96	61.00	98.00	74.00
Influenza	3008	96.99	89.40	84.87	3.01	10.60	15.13	36.73	81.00	89.00
Tuberculosis	4101	92.35	69.09	92.93	7.65	30.91	7.07	26.00	67.00	94.00
Hand-foot-and-mouth disease	1385	75.20	61.68	58.31	24.80	38.32	41.69	26.00	71.00	87.00
Syphilis	855	95.62	90.69	94.24	4.38	9.31	5.76	50.00	86.00	92.00
Infectious diarrhea	525	77.22	61.31	62.06	22.78	38.69	37.94	35.00	93.00	95.00
Scarlatina fever	410	74.14	40.39	69.06	25.86	59.61	30.94	69.00	93.00	94.00
COVID-19	80	93.33	96.00	92.00	6.67	4.00	8.00	54.55	100.00	95.45
Weighted average by test sample size	–	90.44	87.43	86.90	9.56	12.57	13.10	50.26	89.33	80.83

A comparison between the emerging-infectious-disease-prediction accuracies of the three algorithms shows that the performance of LightGBM is better than that of XGBoost, except for viral hepatitis and COVID-19. Furthermore, the infectious-disease-prediction accuracy of LightGBM surpasses that of the Random Forest algorithm, except for tuberculosis. The average prediction sensitivities of LightGBM, XGBoost, and Random Forest are 90.44%, 87.43%, and 86.90%, respectively, indicating that LightGBM is the best algorithm for the proposed model. According to the LightGBM test results listed in Table 7, the accuracy rate of predicting infectious diseases as emerging infectious diseases is substantially higher than the misjudgment rate of non-emerging infectious diseases. Therefore, the second-layer model can reliably distinguish between non-emerging infectious diseases and emerging infectious diseases. The misjudgment rates for predicting hand-foot-and-mouth disease, infectious diarrhea, and scarlet fever as known infectious diseases are relatively high at 24.8%, 22.78%, and 25.86% respectively, but they are also much lower than the rates for correctly predicting emerging infectious diseases, so the model is still capable of providing early warnings for these emerging infectious diseases. The accuracy of EIDDM is listed in Table 8.

**Table 8:** Accuracy rate of the two-layered EIDDM.

Category of infectious diseases	Number of emerging infectious diseases for test	Number of non-infectious disease for test	${TUK}_{2k}$	${A\mathrm{ccuracy}}_{k}$ (%)	${Sensitivity}_{k}$ (%)	${FNR}_{k}$ (%)	${FPR}_{k}$ (%)
Viral hepatitis	23,929	35,894	14,069	82.39	58.79	41.21	1.15
Influenza	3890	5835	2772	86.96	71.26	28.74	0.94
Tuberculosis	5313	7970	3478	83.66	65.46	34.54	1.09
Hand-foot-and-mouth disease	1573	2360	1013	84.80	64.40	35.6	0.42
Syphilis	916	1374	786	92.88	85.81	14.19	1.20
Infectious diarrhea	508	762	356	82.99	70.08	29.92	2.94
Scarlatina fever	409	614	281	84.46	68.70	31.3	3.48
COVID-19	87	131	70	89.91	80.46	19.54	2.08
Weighted average by test sample size	–	–	–	83.47	62.32	37.68	1.14

In summary, the overall false positive rate is relatively low owing to the first layer model already excluding a large number of non-infectious cases. Consequently, the second layer decision-making process avoids the misclassification of non-infectious diseases as emerging infectious diseases. The mean value of the overall accuracy is 83.47%. In conclusion, the first layer model TextCNN-Attention and the second layer model LightGBM demonstrate strong discriminative capabilities for distinguishing between infectious and non-infectious diseases, as well as between non-emerging infectious diseases and emerging infectious diseases.

### Model classification threshold

The model prediction threshold (set to 0.5 in this study) significantly influences the prediction results, and thus, different thresholds are set to evaluate the prediction performance. We increase the threshold of the one-to-many LightGBM model to 0.4 and 0.6, and compare the resulting prediction performance with that observed at a threshold of 0.5. The corresponding results are shown in Table 9.

**Table 9:** Test results obtained when the threshold of the second layer LightGBM OvR model is set to 0.4 and 0.6.

Category of infectious diseases	The number of infectious diseases predicted by the first-level model(TP_1K_ + FP_1k_)	Threshold is 0.4			Threshold is 0.6		
		Sensitivity_2k_	FNR_2k_	FPR_2k_	Sensitivity_2k_	FNR_2k_	FPR_2k_
Viral hepatitis	16,207	87.44	12.56	58.79	94.57	5.43	63.52
Influenza	3008	96.82	3.18	26.67	92.39	7.61	44.67
Tuberculosis	4101	90.43	9.57	22.69	85.94	14.06	28.66
Hand-foot-and-mouth disease	1385	69.25	30.75	15.79	77.24	22.76	39.47
Syphilis	855	93.15	6.85	33.33	92.58	7.42	66.67
Infectious diarrhea	525	71.86	28.14	23.44	70.38	29.62	46.88
Scarlatina fever	410	70.36	29.64	51.61	70.16	29.84	74.19
COVID-19	80	92.00	8.00	20.00	91.25	8.75	80.00
Weighted average by test sample size	–	87.64	12.36	45.60	91.16	8.84	54.74

Tables 7 and 9 suggest that when the threshold is 0.5, the average sensitivity, average false negative rate, and average false positive rate of emerging infectious diseases are 90.44%, 9.56%, and 50.26% respectively. When the threshold is 0.4, the average sensitivity, average false negative rate, and average false positive rate are 87.64%, 12.36%, and 45.60% respectively. We can see that when the threshold is lowered, the sensitivity as well as the false positive rate will decrease. When the sensitivity and the false positive rate decrease concurrently, this study chooses to retain the model with higher sensitivity; when the threshold is equal to 0.6, the average sensitivity is 91.16%, the average false negative rate is 8.84%, and the average false positive rate is 54.74%. We can see that the average sensitivity has increased by less than 1 percentage point, but the average false positive rate has increased by more than 4 percentage points. Therefore, the final threshold in this study was set at 0.5.

### Results of spatial cluster analysis by Knox method

As indicated before, the EIDDM can effectively distinguish between infectious and emerging infectious diseases. However, the monitoring and early warning of infectious diseases also require spatiotemporal information. In the simple time-aggregation detection method, the time lags when the aggregation is discovered. In contrast, the time–space aggregation analysis uses the complete spatiotemporal data, and its prediction results are more accurate and are obtained in less time. Therefore, among the suspicious people who have been screened for carrying emerging infectious diseases by the EIDDM, it is necessary to identify the existence of a spatial cluster of the suspected emerging infectious diseases based on information such as time of detection of the infectious disease, home address, and work address.

Latent infected individuals play a greater role in transmission in the saturation stage of an epidemic ^44^. The incubation periods of each infectious disease analyzed in this study are shown in Table 10. The calculated test statistics $X$ and expectation $E$ at different intervals of *D* (200, 500, 1000, and 2000 m) are represented in Table 11, which shows many case pairs of influenza, viral hepatitis, and tuberculosis. Therefore, cluster analysis is conducted on 1000 cases of partial viral hepatitis that occurred in 2019, 500 cases of partial influenza that occurred in 2019, and 737 cases of tuberculosis that occurred in 2019.

**Table 10:** Incubation period of different infectious diseases.

Category of infectious diseases	Influenza	Hand-foot-and-mouth disease	Viral hepatitis	Syphilis	Tuberculosis	Scarlatina fever	Infectious diarrhea	COVID-19
Incubation	4	7	30	14	25	4	8	10

**Table 11:** Test statistics $X$ and expectation $E$ of infectious diseases at different distances during the incubation period.

Category of infectious diseases	D = 200 [X,E]	D = 500 [X,E]	D = 1000 [X,E]	D = 2000 [X,E]
Influenza	[35 ,29.50]	[141, 115.46]	[417, 359.73]	[1255, 1126.88]
Hand-foot-and-mouth disease	[10 ,4.63]	[22 ,15.30]	[65 ,47.66]	[189 ,159. 19]
Viral hepatitis	[73 ,58.43]	[102 ,84.19]	[198 ,180.47]	[560 ,520.52]
Syphilis	[3 ,0.91]	[4 ,1.61]	[7 ,2.68]	[14 ,7.07]
Tuberculosis	[14, 6.52]	[19 ,9.92]	[29, 20.85]	[71, 63.39]
Scarlatina fever	[1, 0.24]	[1, 0.53]	[4, 1.60]	[9, 5.85]
Infectious diarrhea	[2,0.69]	[7,2.34]	[11,6.62]	[33,21.85]
COVID-19	[0, 0]	[0, 0]	[2, 0.43]	[7, 2.77]

When $X$ = 0, there is no statistical significance, and the corresponding *P*-values are replaced by "-" in Table 12.

**Table 12:** *P*-values of spatial and temporal aggregation of infectious diseases.

Category of infectious diseases	D = 200	D = 500	D = 1000	D = 2000
Influenza	0.175	0.044	0.04	0.073
Hand-foot-and-mouth disease	0.039	0.108	0.041	0.152
Viral hepatitis	0.036	0.032	0.15	0.104
Syphilis	0.086	0.075	0.022	0.021
Tuberculosis	0.008	0.009	0.081	0.307
Scarlatina fever	0.34	0.612	0.306	0.173
Infectious diarrhea	0.161	0.014	0.099	0.048
COVID-19	—	—	0.056	0.028

The null hypothesis is that there is no spatiotemporal cluster of emerging infectious diseases. Table 10 shows that the test statistics *X* are greater than their expected E, and the significance level of the usual hypothesis is 0.05. According to Table 12, except for scarlet fever, the *P*-values of all the emerging infectious diseases are less than 0.05. Accordingly, the null hypothesis is rejected; that is, except scarlet fever, the other emerging infectious diseases are likely to accumulate in time and space ^45^. By comparing the *P* -values with the calibration level of 0.05, it can be concluded that the spatial clusters of influenza occurs at 500 and 1000 m, those of the hand-foot-mouth disease occurs at 200 and 1000 m, and that of the viral hepatitis occurs within 500 m. In the case of COVIS-19, the spatial cluster occurred at 2000 m, indicating that although the new coronavirus has a strong infectious ability, the distance of 2000 m obtained in this study is a result of the controlled epidemic prevention policy of China. High-frequency nucleic acid screenings and a timely sealing and control prevent large-scale infections ^46,47^. Scarlet fever is within the range of 200, 500, 1000, and 2000 m, and its *P*-value is greater than 0.05, which may be caused by the deviation in the distance selection. The test statistic *X* and expectation *E* at the interval of 700 m are recalculated as 3 and 0.87, respectively. The *P*-value is 0.047, which is less than the inspection level by 0.05. These results reveal that all infectious diseases accumulate in time and space within 2 km.

### Test results of the model’s response speed

In the performance test, all the infectious diseases and 1.5 times more non-infectious diseases are used as the training set, and a two-layer model is trained. The test results show that although the training process is computationally intensive, the model requires approximately 27 ms to analyze the case of a new patient . These results were obtained by using an Intel Xeon Gold 5117 CPU 14 core with a 64 Gb memory server under the Keras framework, which can aid in achieving a good response speed. Table 13 shows the performance test results of the model service. The test results of this study are compared with those of our previous MIDDM study ^24^, in which we used the one-hot coding method to classify the infectious diseases.

**Table 13:** Performance test results of different concurrent models.

Concurrency number	EIDDM				MIDDM			
	100	200	500	1000	100	200	500	1000
Average(ms)	230	1278	4402	11,146	467	2411	7889	18,562
Median(ms)	176	1277	3912	7179	285	2355	7628	9831
90% Line(ms)	453	2275	8728	24,324	765	3564	15,638	31,561
95% Line(ms)	647	2342	9144	32,765	810	3624	16,423	36,577
99% Line(ms)	1140	2854	9367	33,469	1457	3988	17,004	37,021
Min(ms)	87	130	160	110	143	186	210	193
Max(ms)	1263	2957	15,346	33,925	1588	4105	18,566	38,820

## Discussions

### Comparison and discussion of methods

The method proposed in this study, utilizing the TextCNN deep learning model, differs from the decision tree and naive Bayes single models employed by Prilutsky et al.^48^. The TextCNN model incorporates various convolutional layers to extract text features, enabling it to capture semantic characteristics at multiple levels. Furthermore, the model includes a multi-head self-attention mechanism, which assigns specific attention weights to features, resulting in more precise classification. To compare various models, we conducted a replacement experiment in the first layer model, where we used LSTM. The results are presented in Table 14. Based on the experimental comparison between the first layer TextCNN-Attention model and the LSTM model, we can observe that the LSTM model exhibits, on average, approximately 4% lower sensitivity and accuracy compared to the TextCNN-Attention model. In other words, the TextCNN-Attention model demonstrates better performance.

**Table 14:** LSTM results for various infectious diseases.

Category of infectious diseases	Number of emerging infectious diseases for test	Number of non-infectious disease for test	${Sensitivity}_{1k}$	${Specificity}_{1k}$	${PPV}_{1k}$	${NPV}_{1k}$	${Accuracy}_{1k}$
Viral hepatitis	23,929	35,894	60.84	94.05	87.21	78.27	80.77
Influenza	3890	5835	70.16	91.62	84.81	82.16	83.04
Tuberculosis	5313	7970	68.81	92.28	85.59	81.61	82.89
Hand-foot-and-mouth disease	1573	2360	80.53	95.41	92.12	88.03	89.46
Syphilis	916	1374	82.72	90.56	85.38	88.71	87.42
Infectious diarrhea	508	762	82.95	84.63	78.25	88.16	83.96
Scarlatina fever	409	614	85.76	90.03	85.14	90.47	88.32
COVID-19	87	131	81.31	92.24	87.44	88.14	87.88
Weighted average by test sample size	–	–	65.01	93.33	86.74	80.14	82.00

The second layer model, LightGBM, is an ensemble framework implementing the Boosting algorithm idea, combining multiple learners to achieve better generalization compared to individual learners. In contrast to disease probability graphs^23^ used for predicting emerging contagious diseases, the knowledge-graph-based approach heavily relies on prior knowledge and domain experts' experiences rather than direct learning from complete patient medical record data. This approach may be limited by data quality and completeness, leading to less comprehensive and accurate predictions. Furthermore, constructing knowledge graphs typically requires manual curation and annotation by domain experts, demanding substantial human resources and time for data collection, labeling, and validation. In contrast, machine learning models based on medical records utilize large-scale real-world healthcare data, enabling automatic learning of patterns and correlations without the need for extensive human intervention. We are currently testing integrated models such as LightGBM and XGBoost based on the Boosting strategy and Random Forest based on the Bagging strategy. In the future, we will consider further validating our experiments on linear and nonlinear models such as SVM^49^ as well as similarity-based models such as KNN^50^.

In recent years, major achievements have been made in Natural Language Processing (NLP) tasks through the use of large language models, such as the GPT model series introduced by OpenAI (e.g., GPT-2 and GPT-3) ^51,52^, and Google's BERT model^53^. These models often exhibit remarkable performance owing to their vast number of parameters, reaching billions, bestowing them with powerful language representation capabilities. However, these achievements also come with certain challenges and limitations. BERT and large language models require substantial computational resources and storage space for training and inference and also necessitate substantial investments in time and computational power for pre-training or fine-tuning. In contrast, TextCNN, combined with Attention, typically possesses a smaller model size and computational complexity, enabling model training under less resource-intensive conditions, while also providing faster response times during prediction.

In this study, the Knox method was employed because of its relatively simple principles, fast computation, and ease of result interpretation and explanation. Furthermore, Knox is a non-parametric method that does not assume data adherence to specific distributions, making it effective even when data distribution is unknown or does not follow specific distribution assumptions. Knox is also less sensitive to irregularly shaped geographic regions, ensuring that the analysis is not influenced by arbitrary geographical boundaries. Therefore, Knox can serve as an exploratory tool to detect potential spatial and temporal clusters in infectious disease data. Its convenience allows for quick and preliminary assessments, aiding researchers in deciding whether further investigation is warranted. The Knox method used in this study enables rapid monitoring of clustering within a 2-km range, providing more specific information compared to nationwide large-scale monitoring, and can identify areas of high priority for further investigation.

### Unbalanced dataset processing

In this study, we first extracted all outpatient, emergency, and hospitalization data for ten years from January 1, 2012 to December 31, 2021. Therefore, the number of samples of non-communicable diseases was quite large. Considering the running time and storage issues, we first use the down-sampling method and select 1.5 times the total number of known infectious diseases as the initial enrollment samples of non-communicable diseases. However, owing to the large difference in the number of samples of various known infectious diseases, the issue of data imbalance for the first- and second-layer training samples in the experiment persists. In order to further improve this characteristic of the content, we can consider optimizing the following aspects in the future:

For minority samples, oversampling methods could be used to increase the sample size of minority categories, such as SMOTE^54^, ADASYN^55^, or Borderline-SMOTE^56^.

For most samples, over-sampling methods could be used to reduce the number of samples in most categories, such as Random Under sampling, Cluster Centroids, or Tomek Links.

### Limitations

In this study, we developed a suitable model and used it to predict emerging infectious diseases by using medical records, time information, and spatial information as well as through a correlation analysis of the symptoms shown by doctors and patients. However, the proposed model exhibits several limitations as well: (1) The data sources used in this study were primarily sourced from several medical institutions, which can cover the epidemic situation in some areas of Beijing, China; however, it is still necessary to obtain information from cross-provincial medical institutions and further optimize the model. (2) The quality of medical records is affected by the writing style of the doctors, and some symptom results are expected to be missing from the medical records; these two factors affect the accuracy of the model. (3) The developed model, EIDDM, performed poorly when tuberculosis, syphilis, and such type of infectious diseases were used as the test data (medical records); therefore, because of misjudgment of non-infectious diseases or poor quality of medical records, the extracted characteristics are not accurate; this shortcoming needs to be mitigated via further optimizations in the future. (4) Owing to its primary reliance on convolutional operations to capture local features in text, TextCNN may exhibit insensitivity to certain text tasks that involve explicit word order and long-range dependencies. In contrast, BERT and large language models excel in modeling global semantics and capturing semantic relationships within the text through self-attention mechanisms. Therefore, in the future, when computational resources and storage space permit, further experimentation should be conducted to explore the feasibility of employing large language models and fine-tuning BERT for the task of infectious disease prediction. This approach is likely to enhance the modeling of complex textual patterns and semantic relationships, potentially leading to improved predictive performance in such tasks. (5) The association analysis of infection symptoms of medical personnel, proposed in this study, cannot be verified, because the original data that were clinically reviewed and determined as the transmission chain did not contain real medical data. Future studies should be designed to incorporate data from multiple medical institutions and to focus on the transmission chain data of infection symptoms by further verifying and optimizing the model attributes.

## Conclusion

The emerging-infectious-disease-prediction framework proposed here is the first ever reported method based on the analysis of real and complete medical records. In this study, we used a hierarchical model and the Knox method to experimentally realize early warning and monitoring of emerging infectious diseases by using the complete medical records obtained medical institutions. The findings of this study prove that emerging infectious diseases can be monitored using the proposed model framework, which incorporates real medical records sourced from medical institutions.

## Data Availability

The data that support the findings of this study are available from the corresponding author upon reasonable request.
